# Exostosis, with Loss of Tooth: A Case in Practice

**Published:** 1903-06

**Authors:** George T. Baker

**Affiliations:** Boston, Mass.


### EXOSTOSIS, WITH LOSS OF TOOTH: A CASE IN PRACTICE.

BY GEORGE T. BAKER, D.D.S., BOSTON, MASS.

In November, 1897, Miss C., aged thirty-nine, experienced very severe pains in the left superior maxillary region. The pain usually began about six o'clock in the afternoon and lasted until bedtime. While very severe, it was not sufficiently localized to enable her to state exactly where it was, but it seemed to be in the vicinity of the upper left bicuspids or molars. This pain continued every day for two or three weeks. She supposed it to be "neuralgia," and at the end of the third week it disappeared without treatment, and complete immunity was enjoyed until the beginning of August, 1898, a period of about nine months. It then began, more severe than before, generally commencing at bedtime and continuing two or three hours, when patient would sleep and rise in the morning free from pain.

October 1, 1898, she consulted me regarding it, stating the above facts and adding that she was very anxious to save all her teeth, and she hoped that not even one would have to be sacrificed.

On examination, nothing was found in the mouth to account for the unusual condition, except that the upper left second bicuspid contained on its occluso-distal surface an oxyphosphate filling partially dissolved. The gum of normal appearance was apparently healthy, but this tooth alone seemed to respond somewhat to gentle tapping. Believing that this tooth was the cause of the long-continued disturbance, the partially dissolved filling was removed and the bur carried through the thin layer of dentine directly into the pulp-chamber, with but very little pain and but slight hemorrhage. Suspecting pulp-nodules, arsenic was applied, one-fiftieth of a grain, with an equal quantity of sulphate of morphia and enough campho-phénique to make a thin paste. This was allowed to remain in the tooth for one week, during which time patient experienced no trouble. A broach was then passed to the foramen and a thin ichorous fluid was discharged, but the suspected pulp-nodules were not found.

A few days later the pain again returned, and, hoping to break up a possible pus sac, a drill was passed through gum and process to the end of the root. Thinking that this would give relief, the patient was dismissed, but next day, reporting no abatement of the pain, the tooth was extracted and very extensive exostosis was found, extending all over the upper half of the root, causing the tooth to appear larger at the apex than at the neck.

There was some discomfort for about twenty-four hours, after which the pain ceased.

Nearly all the other teeth are in position, and no other has been similarly affected up to the present time, now over five years.

In conclusion, attention is called to the salient points of this case, which may possibly be of help in diagnosing other similar cases:

1. Intermittent character of pain, with paroxysms at night and immunity during the day.
2. Absence of all external indication of trouble, such as swelling, inflammation, or marked sensitiveness to heat or cold.
3. Inability of patient to localize the pain, even when so severe as to banish sleep.
4. Crown of affected tooth had been broken away, so that there was no occlusion with lower teeth.

